# Knockout of an outer membrane protein operon of *Anaplasma marginale* by transposon mutagenesis

**DOI:** 10.1186/1471-2164-15-278

**Published:** 2014-04-11

**Authors:** Francy L Crosby, Heather L Wamsley, Melanie G Pate, Anna M Lundgren, Susan M Noh, Ulrike G Munderloh, Anthony F Barbet

**Affiliations:** 1 College of Veterinary Medicine, University of Florida, Department of Infectious Diseases and Pathology, 2015 SW 16th avenue, Gainesville, FL 32610, USA; 2 Physiological Sciences, 2015 SW 16th avenue, Gainesville, FL 32610, USA; 3 USDA-ARS Animal Disease Research Unit, Animal Disease Research Unit, 3003 ADBF, Pullman, WA 99164, USA; 4 Department of Entomology, University of Minnesota, 219 Hodson Hall 1980 Folwell avenue, St. Paul, Minneapolis, MN 55108, USA

## Abstract

**Background:**

The large amounts of data generated by genomics, transcriptomics and proteomics have increased our understanding of the biology of *Anaplasma marginale*. However, these data have also led to new assumptions that require testing, ideally through classical genetic mutation. One example is the definition of genes associated with virulence. Here we describe the molecular characterization of a red fluorescent and spectinomycin and streptomycin resistant *A. marginale* mutant generated by *Himar1* transposon mutagenesis.

**Results:**

High throughput genome sequencing to determine the *Himar1*-*A. marginale* genome junctions established that the transposon sequences were integrated within the coding region of the *omp10* gene. This gene is arranged within an operon with *AM1225* at the 5’ end and with *omp9*, *omp8*, *omp7* and *omp6* arranged in tandem at the 3’ end. RNA analysis to determine the effects of the transposon insertion on the expression of *omp10* and downstream genes revealed that the *Himar1* insertion not only reduced the expression of *omp10* but also that of downstream genes. Transcript expression from *omp9*, and *omp8* dropped by more than 90% in comparison with their counterparts in wild-type *A. marginale*. Immunoblot analysis showed a reduction in the production of Omp9 protein in these mutants compared to wild-type *A. marginale*.

**Conclusions:**

These results demonstrate that transposon mutagenesis in *A. marginale* is possible and that this technology can be used for the creation of insertional gene knockouts that can be evaluated in natural host-vector systems.

## Background

*Anaplasma marginale* is a tick-borne and obligate intracellular bacterium that causes bovine anaplasmosis, a disease that has gained particular attention due to the considerable economic losses for the cattle industry [1-4]. Onset of clinical disease is mainly characterized by a severe hemolytic anemia [1,2]. Cattle that survive acute infection become carriers of *A. marginale* and organisms can be transmitted to susceptible cattle mechanically or by tick bite [2]. *A. marginale* persists in carrier cattle because of its capability to subvert the immune system using antigenic variation in which different variants of outer membrane proteins such as Msp2 and Msp3 are expressed [5-8].

Work on the development of a preventive vaccine against this disease began in the early 1900’s with the isolation of *A. marginale* subsp. *centrale*[9,10]. This less virulent strain, originally from South Africa, is used for immunization of cattle in Africa, Australia, South America and the Middle East and remains the most widely-used and practical vaccine against bovine anaplasmosis [9-11]. This vaccine is not approved in the United States because of the risk of transmitting contaminant blood-borne pathogens that will infect cattle [1]. Recently, comparative genomic studies demonstrated that proteins that are conserved in US strains were not conserved in *A. marginale* subsp. *centrale*[10-12].

Different vaccination methods have been developed for the control of bovine anaplasmosis that range from attenuated live or killed organisms, to DNA and recombinant protein vaccines [9]. But *A. marginale* derived from cell culture, killed organisms and DNA vaccines induce only partial protection [13-15]. Immunization trials using outer membrane proteins or a complex of linked or unlinked outer membrane proteins of *A. marginale* derived from erythrocytes have demonstrated good protection against high bacteremia, anemia and homologous strain challenge [16-20]. However, to promote long lasting protection, several immunization boosts may be required and in addition to this, production and purification of these components is time-consuming and expensive.

The increased use of molecular approaches such as whole genome, RNA sequencing, proteomics and comparative genomics of *A. marginale* has identified potential virulence-associated targets that can be altered or removed by reverse genetics techniques [12,21-25]. This could allow the creation of attenuated organisms that have reduced pathogenicity but still elicit cellular and antibody responses that stimulate immunity without causing disease. Consequently the development of genetic tools to transform *A. marginale* and generate *in-vitro* gene knockouts, or insertional mutants that can be tested for attenuation in their *in-vivo* environment is of great significance.

One way to create insertional mutations in pathogenic bacteria is via transposon mutagenesis, in which a library of recombinant bacteria containing different transposon insertions can be created, allowing for the screening of mutant strains with diverse phenotypes [26,27]. The *Himar1* transposon is a non-replicative class II DNA transposon that is a member of the Tc1/mariner family and is often used for the creation of insertional mutants. Since these types of transposons are horizontally transferred between species, they do not have host restricted functions, making them suitable for use in a wide-range of eukaryotic and prokaryotic hosts [27,28]. In addition to this, the *Himar1* transposon does not have DNA target specificity since it is integrated randomly in TA dinucleotide sites [28-30]. Because of these advantages, transposon mutagenesis using this system has been successfully developed in other tick-borne pathogens such as *Rickettsia rickettsii*, *Coxiella burnetii*, *Borrelia burgdorferi*, *Francisella tularensis*, *Ehrlichia chaffeensis* and *Anaplasma phagocytophilum*[31-40]. These previous results suggest that this system could be useful for the transformation of *A. marginale*.

Nevertheless, previous attempts to transform *A. marginale* by transposon mutagenesis were not successful. Previously, the *Himar1* transposon and transposase were delivered in two separate vectors into *A. marginale* which resulted in the isolation of green fluorescent and antibiotic resistant bacteria. However molecular characterization of these recombinant organisms established that the entire plasmid carrying the transposon sequences was integrated into the *A. marginale* chromosome by a single crossover homologous recombination mechanism instead of the classical cut and paste mechanism of transposition [41]. Therefore, we wanted to evaluate first, if classical transposon mutagenesis using the *Himar1* transposon system is achievable in *A. marginale*, and second, if transposon mutagenesis using this system, is useful for the creation of insertional knockout mutations.

## Results

### Transformation of *Anaplasma marginale* by transposon mutagenesis

Attempts to transform *A. marginale* by transposon mutagenesis using the *Himar1* transposon/transposase system delivered in two separate plasmids were not successful. The probability that two plasmids are introduced at once into *A. marginale* organisms could be very low, especially when viability in the extracellular environment might be highly compromised, resulting in a low fraction of cells competent to take up DNA.

**Figure 1:**
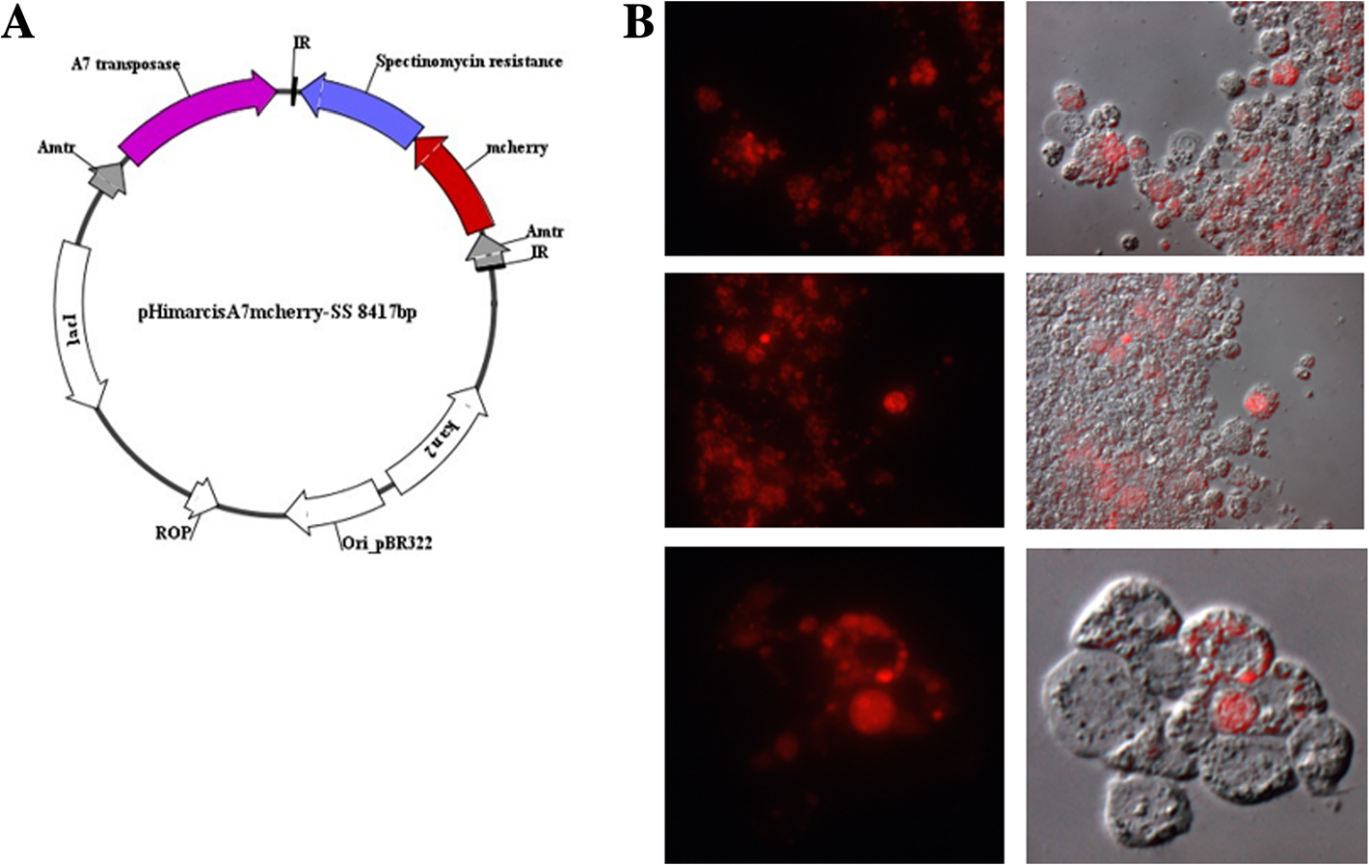
**Red fluorescent *A. marginale*. A**. Plasmid map of *pHimarcisA7mCherry-SS* used for the electroporation of *A. marginale* str. Virginia. **B**. Fluorescent (left) and phase contrast/fluorescence merged (right) images, of transformed *A. marginale* replicating in ISE6 tick cells.

Therefore in order to promote transposon mutagenesis in these bacteria, the transposase was provided in *cis* with the *Himar1* transposon sequences (R. F. Felsheim unpublished data). The *pHimarcisA7mCherry-SS* contains the hyperreactive allele *A7* transposase and the *Himar1* TIR flanking the *mCherry* reporter gene and the *aadA* gene, which confers resistance against spectinomycin and streptomycin. Expression of the transposase and the reporter and antibiotic selection genes is driven by the *A. marginale tr* promoter [41,42] (Figure 1A). Antibiotic selection pressure of electroporated bacteria with this construct resulted in the isolation of red fluorescent and antibiotic resistant bacteria (Figure 1B).

### Mapping of transposon insertion within the *A. marginale* chromosome

We used Roche/454 and Illumina high-throughput genome sequencing to determine: 1) the location of plasmid sequences within the *A. marginale* chromosome, 2) the recombination mechanism that allowed the segregation of mutant bacteria and 3) if these recombinant organisms correspond to a population containing insertions in different genomic locations or in a single genome site.

Mutations produced by the integration of the *Himar1* transposon into the *A. marginale* chromosome will generate new junction sequences that are absent in the wild-type. These new sequences should include the *Himar1* terminal inverted repeats (TIR) followed by the sequence of the regions in which the transposon is integrated. Based on this, the strategy that we used to map the *Himar1* insertion site involved alignment of the sequencing reads obtained by Roche/454 and Illumina methods to two reference sequences, the *A. marginale* str. St Maries genome sequence (CP000030) and the *Himar1* TIR sequence. The *Himar1* TIR-*A. marginale* genome junctions were identified by extracting reads that aligned to the *A. marginale* genome at one end and to the *Himar1* TIR at the other end.

**Figure 2:**
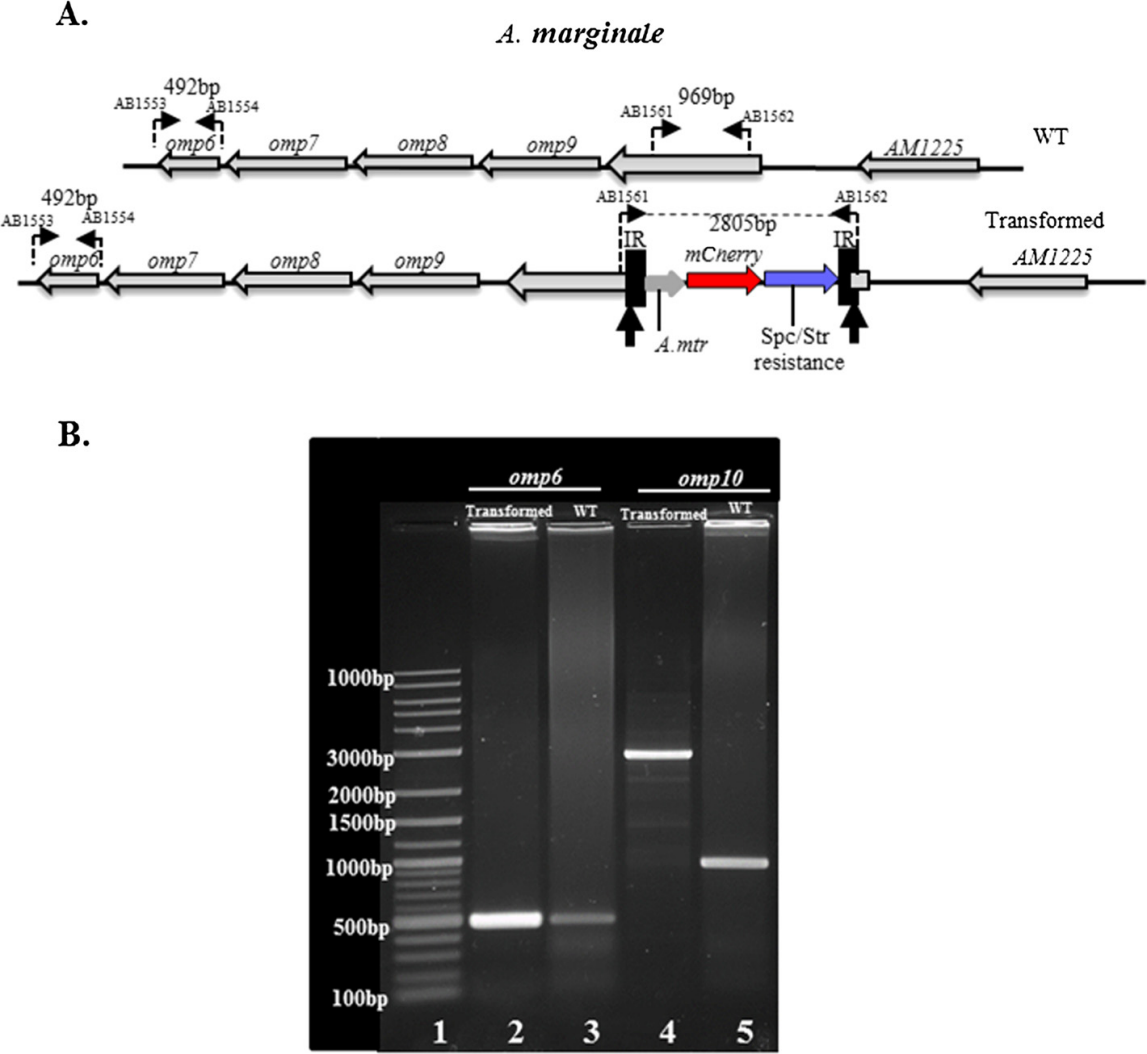
**Mapping of *Himar1 *transposon insertion site. A**. Location of primer pairs (AB1553-AB1554) and (AB1561-AB1562) designed to target the *omp6* and *omp10* genes respectively, in wild-type (WT) and transformed *A. marginale*. Based on sequencing results the *Himar1* sequences are integrated in the chromosome of transformed *A. marginale* at nucleotide 245 after the first base of the *omp10* start codon (arrows) and *mCherry* and *aadA* (Str/Spc resistant) genes are in the opposite orientation to *omp10*. **B**. Agarose Gel electrophoresis. gDNA isolated from ISE6 tick cells infected with wild-type (WT) and transformed *A. marginale*, was used as template for PCR amplification with primers shown in A. (Lane 1) 100 bp/1Kb DNA ladder, *omp6* amplicons in transformed (lane 2) and WT (lane3) *A. marginale* were of the same size 492 bp. The *omp10* amplicon in transformed *A. marginale* (lane 4) was 2805 bp, while in wild type was 969 bp (lane 5).

Analysis using Illumina reads mapped the *Himar1* TIR-*A. marginale* genome junctions into a region of *omp6* and *omp10* genes. Interestingly these reads contained the same mutated sequence. The *omp6* and *omp10* genes share a large stretch of identity 456 nt/459 nt (99%) [43]. The short, 100 nt length of the Illumina reads, made it difficult to differentiate which gene contained the *Himar1* transposon. Additional analysis using longer reads obtained on the Roche/454 platform revealed that the *Himar1* transposon was integrated within the *omp10* gene. These reads contained a region of *omp10* that is not shared with *omp6*. Based on this sequencing analysis the genomic location of the *Himar1* transposon in the chromosome of the transformed *A. marginale*, is at position 245 considering 1 as the first base of the *omp10* start codon (Figure 2A).

These results were verified by PCR amplification of gDNA from ISE6 cells infected with wild-type and transformed *A. marginale* using *omp6* and *omp10* specific primers (Figure 2A-B). The size of *omp6* amplicons (492 bp) in wild-type and transformed *A. marginale* was the same. However the size of the *omp10* amplicon in transformed *A. marginale* was increased by 1836 bp when compared to the wild-type (969 bp), indicating that the transposon was integrated within the *omp10* gene.

**Figure 3:**
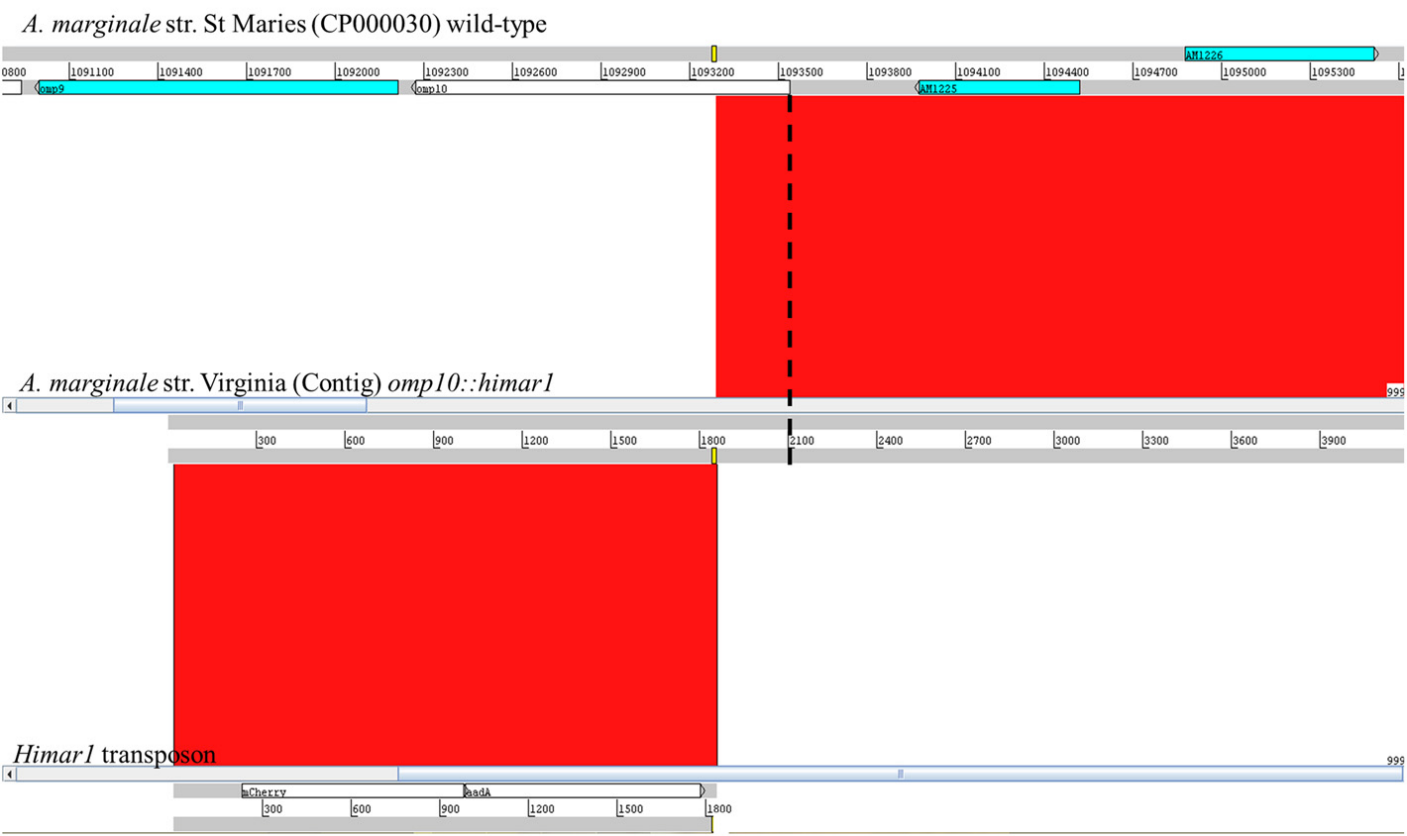
***Himar1 *transposon insertion site in the *A. marginale *str. Virginia genome.** ACT (Artemis Comparison Tool) window showing alignment between the *A. marginale* genome (CP000030) used as reference, the *A. marginale* str. Virginia *omp10::himar1* contig formed by Roche/454 and Illumina sequencing reads and the *Himar1* transposon sequences. Alignment between the *A. marginale* str St Maries and the *omp10::himar1* mutant shows that sequences flanking the transposon insertion site are highly similar sharing an identity of 99% (matching red band). This demonstrates that the transposon insertion site (yellow boxes) occurred at nucleotide 245 after the first base of the *omp10* start codon (black dotted line) in the reference strain. Alignment with the *Himar1* transposon sequences clearly show the insertion of these sequences in the *omp10::himar1* mutant which are not present in the *A. marginale str*. St Maries (absence of matching band).

The genome sequence of *A. marginale* str. Virginia is available only as unannotated contigs with gaps. Therefore for our analysis we used the *A. marginale* str. St Maries genome as reference. For this reason we wanted to confirm that the transposon location in the mutated Virginia strain was the same as the one mapped in the reference genome. For this, combined Roche/454 and Illumina reads were assembled and a contig of 21,324 nucleotides identified. Alignment of this contig with the *A. marginale* str. St Maries genome showed that this sequence contained part of *omp10* and upstream genes (99% identity) (Figure 3) and that the transposon insertion site in the *A. marginale* str. Virginia matches the same region mapped using the reference genome.

Further analysis of sequencing reads determined that there is only one transposon insertion in the chromosome of recombinant *A. marginale*. The reads containing the *Himar1* TIR-*A. marginale* junctions aligned to a single genome site. Although these transformed organisms were not cloned, data suggest that they are isogenic for the transposon insertion site within the *omp10* gene.

**Figure 4:**
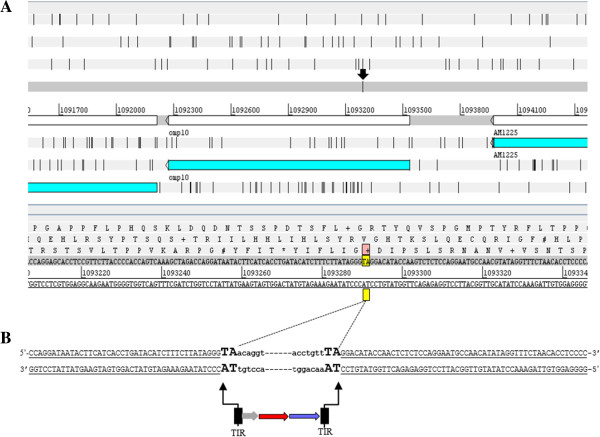
**Characterization of *Himar1 *transposon insertion site. A**. Artemis (genome browser and annotation tool) window showing the *A. marginale* genome (CP000030) used as a reference for the location of the *omp10* gene (*AM1223*, 1092273–1093555), and the TA dinucleotide (1093290–1093291) at the *Himar1* tn insertion site (arrow) determined by high throughput genome sequencing analysis. **B**. *Himar1* tn insertion into the *omp10* gene was mediated by the A7 transposase in a cut and paste mechanism leading to the duplication of TA dinucleotide sequences. *A. marginale* genome (underlined uppercase text, TA dinucleotide duplications (enhanced uppercase text) flanking the tn elements (bold lowercase).

The mobilization of the *Himar1* transposon from one locus to another is mediated by a transposase using a cut and paste mechanism [27,30]. It has been shown previously in other organisms that the *Himar1* transposon integrates preferentially into a TA site and leads to duplication of this dinucleotide upon integration into the target site [30]. This was found to be true also for *A. marginale*. Sequencing analysis revealed that the *Himar1* transposon targeted a TA dinucleotide in *omp10* (Figure 4A) and upon integration it is flanked by a TA dinucleotide sequence (Figure 4B). Thus, the mobilization of the *Himar1* transposon into the *omp10* gene of *A. marginale* was mediated by means of the *A7* transposase in a cut and paste mechanism. This transformant of *A. marginale* will be referred to as *omp10::himar1* mutant.

### Evidence for expression of *omp10* as part of an operon

**Figure 5:**
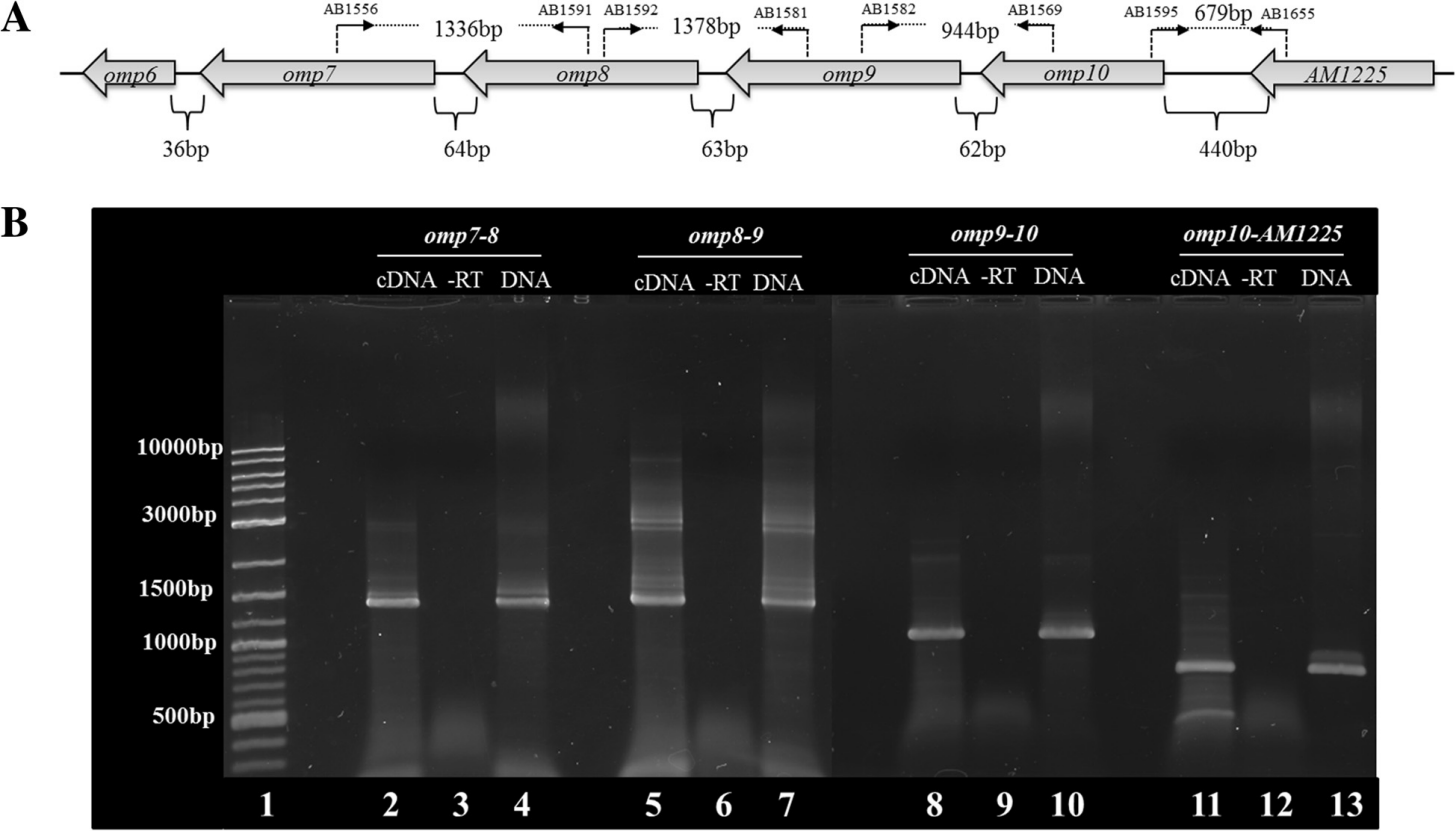
**Intergenic regions of *omp7 *to *AM1225 *were analyzed by RT-PCR. A**. Diagrammatic representation of the *AM1225*-*omp6* operon with *AM1225*, *omp10, omp9*, *omp8*, *omp7* and *omp6* and intergenic regions. Location of primer pairs (AB1556-AB1591), (AB1592-AB1581), (AB1582-AB1569), and (AB1655-AB1595) designed for PCR amplification of *omp7-8*, *omp8-9*,*omp9-10* and *omp10*-AM1225 intergenic regions using cDNA from ISE6 cells infected with *A. marginale* wild-type. **B**. Agarose gel analysis of amplicons connecting intergenic regions from *omp7* through *AM1225* (lanes 2, 5, 8, and 11). DNA was used as positive control (lanes 4, 7, 10, and 13). cDNAs from reactions with no reverse transcriptase were used as negative controls (lanes 3, 6, 9 and 12). 100 bp/1Kb DNA ladder (lane 1).

We hypothesize that the transposon insertion could alter the expression of *omp10* and downstream genes. This hypothesis is based on recent work in which the transcriptome profile of *A. marginale* using RNAseq indicated that *omp10* is expressed as part of a six-gene operon in erythrocytes of infected cattle [25]. This operon includes *AM1225*, *omp10*, *omp9*, *omp8*, *omp7* and *omp6* (Figure 5A).

Because of this, we wanted to determine if *omp10* is expressed within a polycistronic message in *A. marginale* replicating in ISE6 tick cells. The intergenic region between *AM1225* and *omp10* is 440 bp long, while intergenic regions between *omp10-9, omp9-8, omp8-7* and *omp7-6* are 62 bp, 63 bp, 64 bp and 36 bp respectively (Figure 5A). To test whether *AM1225* through *omp7* are expressed as a single transcriptional unit, total RNA isolated from ISE6 cells infected with wild-type *A. marginale* was reverse transcribed and template cDNA was used for amplification of intergenic regions with primers that connect neighboring genes (Figure 5A). The *omp6* gene was not included in these experiments, because previous work [43] and work in our lab showed that transcripts from this gene are not detected in *A. marginale* during infection of tick cells. Appropriate size amplicons of the intergenic regions between *omp7-8, omp8-9, omp9-10* and *omp10*-*AM1225* gene were detected (Figure 5B), providing evidence that these genes are transcribed as a single mRNA in *A. marginale* infected tick cells.

### RNA transcript analysis

**Figure 6:**
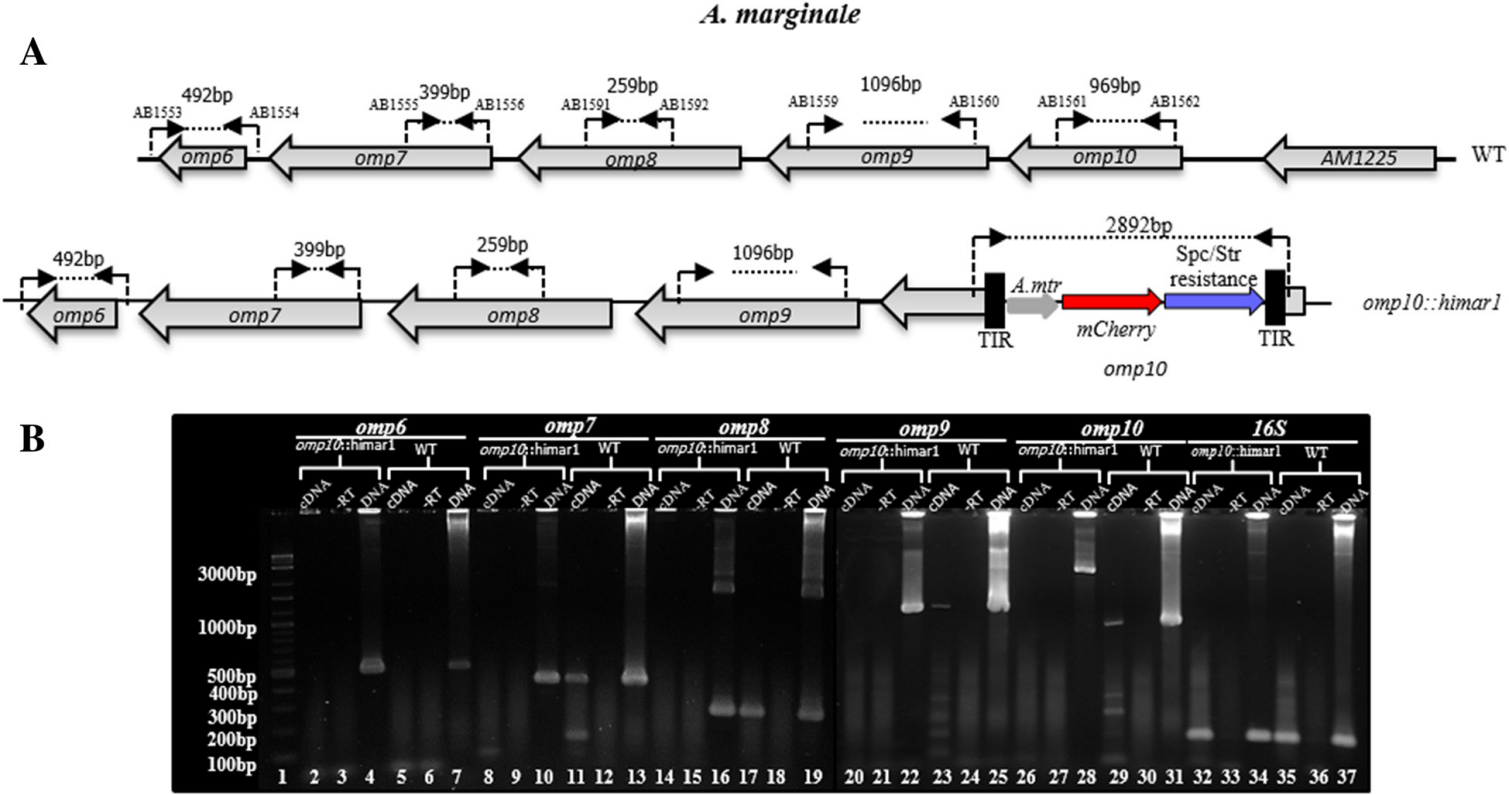
**Transcriptional analysis of the effect of the insertion of the *Himar1 *transposon within the *omp10 *gene by RT-PCR. A**. Binding sites of primers (AB1553-AB1554), (AB1555-AB1556), (AB1591-AB1592), (AB1559-AB1560), and (AB1561-AB1562), designed to amplify transcripts on *omp6, 7, 8, 9* and *10*, respectively, in wild-type (WT) and o*mp10::himar1* mutant. Complementary DNA from WT and o*mp10::himar1* mutant grown in ISE6 tick cells was used for PCR amplification for o*mp6* through *10* with specific primers to evaluate gene expression. **B**. Agarose gel analysis of PCR products for *omp6* through *10* in *omp10::himar1* mutant (lanes 2, 8, 14, 20, and 26). PCR products for o*mp6* through *10* in WT (lanes 5, 11, 17, 23, and 29). Genomic DNA was used as positive control (lanes 4, 7, 10, 13, 16, 19, 22, 25, 28, and 31). Complementary DNA from reactions without reverse transcriptase were used as negative controls (lanes, 3, 6, 9, 12, 15, 18, 21, 24, 27, and 30). 100 bp/1Kb DNA ladder lane 1). *16S* rRNA (AB1572-AB1573) was used as an internal control to ensure integrity of cDNA (lanes 32–37).

Next, we determined if insertion of the *Himar1* sequences resulted in alteration of *omp10* expression and the expression of genes downstream. For this, total RNA from ISE6 tick cells infected with *A. marginale* wild-type and *omp10::himar1* mutant was reverse transcribed and cDNA used as template for PCR amplification with specific primers that were designed to anneal to *omp6, omp7, omp8, omp9, and omp10* in wild-type and *omp10::himar1* mutant respectively (Figure 6A). The *omp10, 9, 8,* and *7* genes, but not *omp6*, are transcriptionally active in wild-type *A. marginale*, although at low levels (Figure 6B). The *Himar1* transposon insertion into the coding sequence of *omp10*, disrupted its expression and that of *omp9*, *omp8*, and *omp7* since transcripts from these genes were not detected in *omp10::himar1* mutants of *A. marginale* by this method (Figure 6B). To ensure integrity, cDNA samples from *A. marginale* wild-type and *omp10::himar1* mutant were used for amplification with specific primers of a region of 131 bp of the *16S* rRNA. Amplicons from this region were detected in both wild-type and *omp10::himar1* mutant. No bands were visualized in negative controls (Figure 6B).

**Figure 7:**
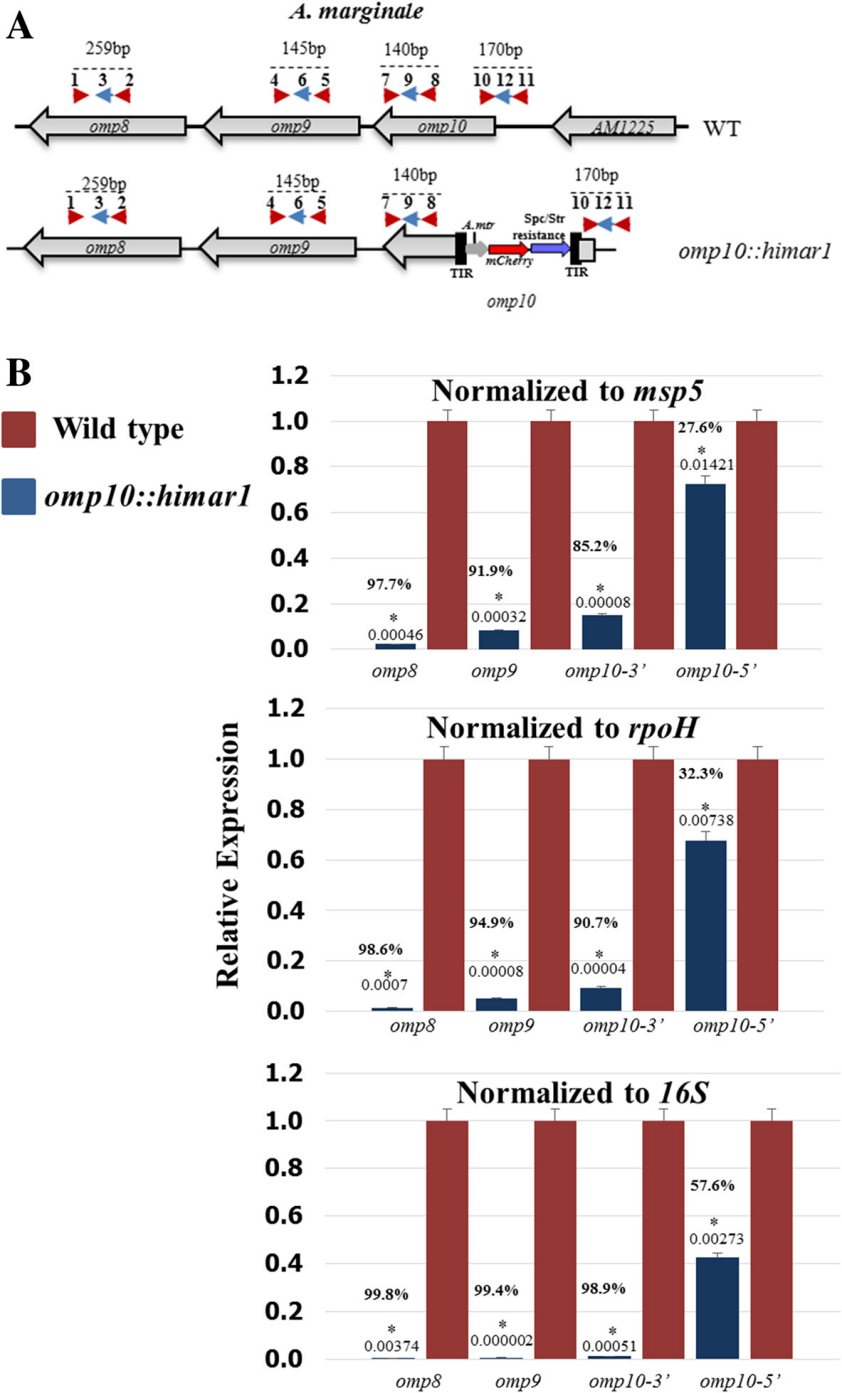
**Relative gene expression by RT-qPCR. A**. location of binding sites for primers and probes designed to target *omp8* (**1**. AB1591, **2**. AB1592, **3**. AB1593), *omp9* (**4**. AB1581, **5**. AB1582, **6**. AB1583), 3’ end of *omp10* (**7**. AB1569, **8**. AB1570, **9**. AB1571), and the 5’ end of *omp10* (**10**. AB1594, **11**. AB1595, **12**. AB1596). **B**. Bar lengths represent the percentage of expression of *omp8*, *omp9*, 3’ end of *omp10* and 5’ end of *omp10* in *A. marginale* wild-type (red bars) and *omp10::himar1* mutant (blue bars). *msp5*, *rpoH* and *16S* rRNA were used as reference genes for data normalization. Changes in expression of these genes were calculated using the 2^-ΔΔCt^method.* Significant differences (P < 0.05) were calculated as described in materials and methods.

Since *omp10* through *omp7* are expressed at low levels in ISE6 tick cells, RT-qPCR was used to quantitatively determine differences of expression between *A. marginale* wild-type and *omp10::himar1* mutant. For this, cDNA generated from ISE6 tick cells infected with *A. marginale* wild-type and *omp10::himar* mutant was used for real time PCR amplification using primers and probes targeting *omp8*, *omp9*, and the 3’ and 5’ ends of *omp10* (Figure 7A).

In order to compare these gene expression results between wild-type and *omp10:himar1 A. marginale*, Ct values were normalized to the *rpoH*, *msp5* and *16S* rRNA genes. Changes in expression of these genes were calculated by the 2^-ΔΔCt^ method, and results were expressed as percentage of expression, with a 100% expression level being assigned to the calibrator or control group, which in this case is wild-type *A. marginale*.

Although three different reference genes were used, RT-qPCR data normalization led to similar results in which there was a significantly reduced expression for *omp8* (97–99%), *omp9* (90–99%) and *omp10 3*’ end (85-98%) relative to their counterparts in wild-type *A. marginale* (Figure 7B). These results show that *Himar1* transposon insertion into *omp10* affected its expression and the expression of genes downstream, confirming the results obtained by RT-PCR and agarose gel electrophoresis. A second experiment investigated the possibility of the same effect occurring in regions of *omp10* before the *Himar1* transposon insertion site. For this, a primer and probe set was designed to anneal with a region at the 5’ end of *omp10* (Figure 7A). Even though there was a significant reduction in the detection of transcripts from this region (27-57%) relative to the 5’ end of *omp10* in wild-type, this reduction was not as great as with the sequences located in *omp10* downstream of the *Himar1* transposon insertion site.

### Western immunoblot analysis

To determine if the decreased expression of mRNA in genes downstream of *omp10* correlated with protein expression a Western immunoblot analysis using anti Omp9 antibody was performed.

To compare the protein expression of *omp9* between *A. marginale omp10::himar1* and wild-type, the number of organisms per sample was quantified by qPCR using the *opag2* single copy gene to determine the copy number of *A. marginale*. Equal amounts (10^8^) of organisms of *A. marginale* wild-type and *omp10::himar1* mutant were loaded per lane. *A. marginale* str. Virginia initial bodies and uninfected ISE6 cells were used as positive and negative controls respectively.

**Figure 8:**
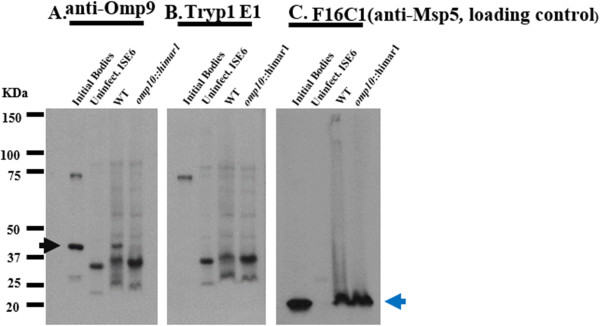
**Immunoblotting of *omp10::himar1 *mutant and WT *A. marginale *using the specific monoclonal antibody Omp9.** Proteins from equal amounts of host cell-free wild-type (WT) and *omp10::himar1 A. marginale* were separated by SDS-PAGE gel electrophoresis. Immunoblot PVDF membranes of transferred proteins were reacted with monoclonal antibodies and reactions were visualized by chemiluminescence. **A**. Monoclonal antibody Omp9 (4 μg/ml) with specificity to Omp9 protein (40 kDa) (black arrow). **B**. Negative control, monoclonal Tryp1E1 (4 μg/ml) (exhibits specificity for a variable surface glycoprotein of *Trypanosoma brucei*. **C**. Monoclonal F16C1 (2 μg/ml), reacts with the Msp5 (19 kDa) (blue arrow) protein of *A. marginale*, was used as loading control. *A. marginale* str. Virginia and uninfected ISE6 cells were used as positive and negative controls respectively.

Western immunoblot showed a reduced expression of Omp9 in *omp10::himar1 A. marginale* mutant compared with wild-type (Figure 8A). The Omp9 band of 40 kDa was present in wild-type and initial bodies but was not detected in the mutant or using negative control antibody Tryp1E1 (Figure 8B). Antibody F16C1 that reacts with major surface protein 5 (Msp5) was used as a loading control. Anti-Msp5 detected this protein (19 kDa) in wild-type and *omp10::himar1 A. marginale* (Figure 8C).

These results correlated with results obtained from the RNA transcript analysis, showing that the transposon insertion severely affected the expression of both mRNA and protein from downstream genes such as *omp9*.

## Discussion

The possibility of creating insertional mutations in *A. marginale* not only could provide a broad understanding of gene products required for infectivity, growth or viability of this pathogen in the mammalian host and the tick vector, but also would allow the generation of genetically attenuated organisms that can be tested in vaccination trials.

Here we report that transposon mutagenesis using the *Himar1* transposon/transposase system for *A. marginale* is achievable and it could be useful for creating insertional mutations in these organisms. High throughput genome sequencing analysis for the characterization of these transformants established that transposon sequences are integrated within the *omp10* gene of the *A. marginale* chromosome and its mobilization within this gene was mediated by the transposase in a cut and paste mechanism, since i.) the transposon sequences were integrated within a TA dinucleotide site ii.) upon integration of the transposon, this sequence was duplicated and is found flanking the transposon TIR at the junctions with the *A. marginale* genome and iii.) sequences from the delivering vector outside the transposon were not found.

Although these *omp10*::*himar1* mutant organisms were not cloned, they are isogenic for the transposon insertion within the *omp10* because all the sequencing reads containing the transposon-*A. marginale* genome junctions aligned to the same genome site in the *A. marginale/St. Maries* reference genome sequence (CP000030). Possible reasons include transposon insertion into other genome regions that are essential for growth in tick cells, or insertion into regions that cause slower growth and non-recovery of these mutants. This suggests that further optimization is required to improve transformation efficiencies and for more rapid identification and separation of mutants before they are visible in cultures.

The *omp10* gene is part of the *omp1* through *omp14* clusters, members of the *msp2* superfamily that correspond to the pfam01617 family of bacterial surface antigens [21]. Deep sequencing of cDNA generated from total RNA of erythrocytes infected with *A. marginale* identified 70 putative operon arrangements. One contained *omp10* transcribed as part of an operon of six genes with *AM1225* at the 5’ end and with *omp9*, *omp8*, *omp7* and *omp6* arranged in tandem at the 3’ end [25]. In order to have a better understanding of the effects of the transposon insertion in *omp10* on adjacent genes it was important to determine if *omp10* is also expressed as part of a polycistronic message in *A. marginale* replicating in tick cell cultures.

RT-PCR of intergenic regions between *omp7-8*, *omp8-9*, *omp9-10* and *omp10*-*AM1225* provided evidence that *omp10* is transcribed within a polycistronic message in *A. marginale* infected tick cells. However transcripts of *omp6* were not detected. Similar results in which *omp6* expression was not detected in *A. marginale* infected IDE8 tick cells and in tick midguts were obtained by others previously [43]. A lack of *omp6* transcripts suggests that this gene may not be expressed in tick cells or only at very low levels. It has been shown that, in bacteria with reduced genomes such as *Mycoplasma pneumoniae*, gene members of an operon are not always expressed at the same levels and those genes distal from the promoter may have lower expression [44].

RT-PCR and relative gene expression experiments demonstrated that insertion of *Himar1* into *omp10* at nucleotide 245 from the start of the ORF altered the sequence of this gene. This resulted in the loss of its expression since there was a significant reduction in the detection of transcripts from this gene when compared with the expression of *omp10* transcripts from wild-type *A. marginale*.

It has been shown that in bacteria production and/or stability of mRNA in regions downstream of a transposon insertion is greatly reduced, to the point where very little mRNA corresponding to this region can be isolated [45]. Insertion of *Himar1* within a gene can affect the expression of neighboring genes, as shown in a variety of bacteria and especially in other tick-borne bacteria [38,39,46]. Therefore, we evaluated the effect of the *Himar1* insertion on the expression of genes downstream and upstream of *omp10* in *omp10::himar1 A. marginale*. Results showed that the transcriptional activities of *omp9* and *omp8* were negatively influenced by the insertion of the *Himar1* within *omp10* since detection of transcripts was significantly decreased in relation to wild-type *omp9* and *omp8*.

Although the transcription activity of regions upstream of the transposon insertion site at the 5’ end of *omp10* dropped significantly in relation to wild-type *A. marginale*, it was not as severe as with genes downstream of *omp10*. Sequencing analysis determined that the transposon sense strand is found in the opposite orientation to *omp10*, so it might be possible for transcription to read through the *Himar1* sequences and produce anti-sense transcripts that could reduce expression of sequences upstream of *omp10*, but to demonstrate this further characterization is required.

Western immunoblot analysis showed that the transposon insertion into *omp10* markedly reduced protein expression of *omp9* in the *omp10::himar1* mutant *A. marginale* when compared to wild-type, corroborating that both mRNA and protein expression from genes downstream of *omp10* were disrupted.

The evidence presented here suggests that these genes are not essential for growth of *A. marginale* in tick cell culture. Significant work on the possible interactions between the expressed proteins in different host environments has accumulated and offers important clues about the possible phenotypic effects of the disruption of these genes in *A. marginale*. For example *omp7*, *omp8*, *omp9* and *omp10* are differentially expressed in tick and mammalian cells with lower levels in tick midgut and cultured tick cells [43]. Detection of proteins from these genes has been reported [43,47,48]. Omp7, Omp8 and Omp9 are conserved during tick transmission and in acute and persistently infected cattle [43]. Characterization of the repertoire of outer membrane surface proteins by mass spectrometry identified Omp10 and Omp7 as immunogenic in cattle [47]. Proteome analysis using crosslinking and liquid chromatography–mass spectrometry (LC-MS/MS) to determine the composition and topological organization of surface proteins in *A. marginale* in mammalian and tick cells isolated a large protein complex and analysis demonstrated that Omp7, Omp8 and Omp9 are arranged in the outer membrane as near neighbors to Msp2, Msp3, Msp4, Omp1, Opag2, Am779, Am780, Am1011, Am854 and VirB1 in *A. marginale* isolated from erythrocytes [18]. In contrast a similar sized large protein complex in *A. marginale* isolated from tick cells was formed only by Msp2, Msp3, Msp4, Am778 and Am854. Although Omp7, Omp8 and Omp9 were expressed they did not seem to be localized to the surface, suggesting a possible re-arrangement in the topology of the surface of *A. marginale* during the transition from the tick cell into the mammalian cell [18].

Interestingly, the number of Msp2 superfamily members such as *omp1* to *omp15* in *A. marginale* subsp. *centrale*, is reduced in comparison with US *A. marginale* strains [10]. For example, closely related sequences to *omp8* and *omp6* are missing and *omp10* is found with *omp7* and a reduced *omp9* in tandem, which may indicate an important function of these genes in the pathogenicity of *A. marginale*.

Based on this, further characterization of these *omp10::himar1* mutants to understand the effects of the disruption of expression of *omp10*, *9*, *8* and *7* on the phenotype of *A. marginale* is of critical importance. Phenotypic effects may include infectivity, tick transmissibility, stability under non selectable conditions, ability to induce immune responses and ability to establish persistent infection within the natural host.

## Conclusions

Transposon mutagenesis is achievable for *A. marginale*. High throughput genome sequencing of recombinant bacteria electroporated with a single plasmid containing the *Himar1* sequences and the *A7* transposase showed insertion of the *Himar1* sequences into the *omp10* gene of *A. marginale*. The insertion was mediated by the transposase in a cut and paste mechanism. In tick cells *omp10* is expressed as a polycistronic message with *AM1225* at the 5’end and *omp9*, *8* and *7* at the 3’ end. Insertion of the *Himar 1* transposon within *omp10* not only disrupted its expression but also the expression of genes downstream, such as *omp9*, *omp8* and *omp7*.

This work shows the utility of the *Himar1* system for the generation of insertional mutants in *A. marginale*, for the identification of genes involved in virulence and potentially for the development of attenuated organisms.

## Methods

### *A. marginale* cultivation

Cultures of *A. marginale* str. Virginia wild-type and *omp10*::*himar1* mutant were maintained in tick ISE6 cells derived from embryonated eggs of the blacklegged tick, *Ixodes scapularis* at 34°C in non-vented 25-cm^2^ cell culture flasks (NUNC). *A. marginale*-infected cell cultures were maintained in L15B300 medium supplemented with 5% fetal bovine serum (FBS, BenchMark, Gemini Bio-Products), 5% tryptose phosphate broth (TPB, Difco, Becton Dickinson), 0.1% bovine lipoprotein concentrate (LPC, MP-Biomedical), 0.25% NaHCO_3_, and 25 mM HEPES buffer, adjusted to pH 7.8, as previously described [49]. The cell culture medium for ISE6 cells infected with the *A. marginale omp10*::*himar1* mutant was supplemented with spectinomycin (Sigma Aldrich) and streptomycin (Sigma Aldrich) to a final concentration of 50 μg/ml each.

### Isolation of the *A. marginale* mutant by transposon mutagenesis

To maximize chances of obtaining a transformant using transposon mutagenesis, we used a single plasmid construct that encoded both the transposon and the transposase in *cis* configuration as described [50], except that the fluorescent marker was replaced by sequences encoding a monomeric red fluorescent protein, *mCherry*[51] (Figure 1A). *A. marginale* bacteria passaged 53 times in ISE6 cells were harvested from one 25-cm^2^ culture in 5 ml of medium when ~80% of cells were infected, and many cells were undergoing lysis. The cells were recovered in 2 ml of culture medium, and added to a 2-ml microcentrifuge tube containing 0.3 ml of sterile silicon carbide abrasive (60/90 grit; Lortone, Inc), vortexed at maximum speed for 30 sec, and the lysate transferred to a fresh 2-ml tube on ice. Bacteria were collected by centrifugation at 11,000 g for 10 min at 4°C, and washed twice in ice-cold 300 mM sucrose. They were then resuspended in 50 μl of 300 mM sucrose containing 3 μg of plasmid DNA, and incubated on ice for 15 min before being electroporated (Biorad Gene Pulser II) at 2 kV, 400 Ohm and 25 μF in a 0.2 cm gap cuvette. The electroporation mixture was recovered in 1.5 ml of an ISE6 cell suspension (~2×10^6^ cells), and centrifuged in a microcentrifuge tube at 1,000 g for 10 min at room temperature. The tube was left undisturbed for 30 min at room temperature, and the pellet then resuspended in the supernatant medium and added to a 25-cm^2^ flask containing ~5×10^6^ ISE6 cells in 3 ml of L15B300 medium supplemented as described for *Anaplasma*-infected cultures. The culture was incubated at 34°C in a tightly capped flask. Three days after electroporation, the culture medium was replaced with 5 ml of medium additionally containing 50 μg/ml of spectinomycin and streptomycin (selection medium). Subsequently, the culture was fed twice weekly with selection medium and examined weekly on an inverted microscope (Diaphot, Nikon) fitted for epifluorescence using a Texas Red filter. The first fluorescent colonies of bacteria were noted 6 wk following electroporation, and the culture was maintained in selection medium with twice-weekly medium changes until ~90% of cells were infected. At that time, the mutant was passaged (ten-fold dilution) to fresh cells, and the remainder was stored in liquid nitrogen.

### Preparation of host cell-free *A. marginale* wild-type and o*mp10*::himar1 mutant from ISE6 tick cells

Isolation of *A. marginale* wild-type and *omp10*::himar1 mutant was performed by disruption of ISE6 tick cells with 1 mm diameter glass beads (BioSpec Technologies) in a Minibead beater (BioSpec technologies) as described elsewhere [52], with the exception that cells were shaken only once for 10s and immediately placed on ice. Cell lysates were transferred to 1.5 ml centrifuge tubes and centrifuged at 100 g for 5 min at 4°C to pellet cell debris. The supernatant was then carefully removed and transferred to clean 1.5 ml centrifuge tubes. *A. marginale* organisms (wild-type and *omp10*::*himar1* mutant) were pelleted at 11,000 g for 10 min at 4°C, and stored at −20°C.

### DNA isolation and Phi29 amplification of the *A. marginale omp10*::*himar1* mutant

Before DNA isolation, pelleted *A. marginale omp10*::*himar1* mutants were treated with RNaseA (QIAGEN) and DNase I (Sigma Aldrich) to remove ISE6 host cell contaminant nucleic acids. DNA isolation was performed using the QIAamp DNA Mini kit (QIAGEN) as per manufacturer’s instructions, but in this case the DNA was eluted in 50 μl of 1 mM Tris pH 9.0. DNA concentration was determined using the Qubit dsDNA HS assay kit (Life technologies) on a Qubit fluorometer (Life technologies). 5 reactions of 10 ng of DNA were used for whole genome amplification using the Genomi Phi V2 DNA amplification kit (GE Healthcare) according to manufacturer’s instructions. Following amplification, aliquots were pooled together and the DNA purified with GelElute Extraction Kit (5 PRIME) by adsorption to silica particles and eluted with 10 mM Tris pH8.2.

### Genome sequencing and bioinformatics

Samples from 2.0 to 3.6 μg of amplified DNA derived from the *omp10::himar1* mutant, were provided for library construction and sequencing by the Roche/454 (GS-FLX) method to the Interdisciplinary Center for Biotechnology Research (ICBR) at the University of Florida. Also, samples of equivalent amounts were provided to the Scripps Research Institute, La Jolla, California for sequencing by the Illumina (HiSeq) method.

A total of 374,151 and 207,288,916 reads of Roche/454 and Illumina sequencing data, respectively, were obtained. The FASTQ files provided by the sequencing facilities were uploaded to the UF GALAXY web site http://galaxy.hpc.ufl.edu, and analyzed separately.

Uploaded Illumina FASTQ files were groomed, filtered and formatted into FASTA files using the FASTQ Groomer, Filter FASTQ and FASTQ to FASTA converter tools located in the NGS: QC and manipulation toolbox of GALAXY. FASTA files were then aligned to the *A. marginale* str*.* St Maries reference genome sequence (CP000030) using the Megablast alignment tool (NCBI BLAST + blastn (version 0.0.12) in GALAXY) to obtain sequencing reads that contained *A. marginale* sequences.

These *A. marginale* sequencing reads were then used for a second Megablast alignment using as a reference sequence 28 nucleotides from the *Himar1* terminal inverted repeats (TIR). The transposon insertion locus within the *A. marginale* chromosome was then determined, since the reads obtained contained the *A. marginale*-*Himar1* TIR junctions.

A similar strategy was used for the analysis of the Roche/454 sequencing reads. CLC genomics workbench, version 6.5 was used for assemblies of Roche/454 and Illumina reads.

### RNA isolation

For RNA isolation, three samples of ISE6 cells infected with *A. marginale* wild-type and three *omp10*::*himar1* samples were used. Each sample derived from separate cultures grown in T-25 cell culture flasks. Samples containing approximately equal numbers of infected cells were collected in RNA stabilization reagent RNAlater (AMBION-Life technologies) and stored at −80°C. Total RNA was isolated using the RNeasy kit (QIAGEN) with an added “on-column” DNase I treatment (QIAGEN) according to manufacturer’s instructions. Aliquots of extracted RNA were used to measure contaminant DNA concentration using the Qubit dsDNA HS assay kit (Life technologies). Additionally, RNA was treated three times with RNase-free Dnase I (AMBION-Life technologies) to remove any trace of contaminant DNA in the sample. RNA concentration was measured with the Qubit RNA assay kit (Life technologies), and samples were stored at −80°C.

### RT-PCR and RT-qPCR experiments

RNA (2 μg) from ISE6 cells infected with *A. marginale* wild-type and *omp10::himar1* mutant was converted to cDNA by random priming using a Omniscript reverse transcriptase kit (QIAGEN) according to manufacturer’s conditions. Genomic DNA and no-reverse transcriptase reactions were included as controls for each sample and each nucleic acid target. Specific primers (Table 1) were designed to amplify transcripts from intergenic regions between *omp7*-*omp8*, *omp8*-*omp9*, *omp9*-*omp10* and *omp10*-*AM1225* using cDNA from ISE6 cells infected with *A. marginale* wild-type as template. Similarly transcripts from within *omp6*, *omp7*, *omp8*, *omp9*, and *omp10* genes were detected by PCR amplification of cDNA from ISE6 cells infected with *A. marginale* wild-type and the *omp10::himar1* mutant using *omp6-10* specific primers (Table 1). PCR amplification conditions for each PCR experiment are described in Additional file 1: Tables S1 and S2 respectively.

**Table 1:** PCR and Taqman qPCR oligonucleotides used in this study

**Oligonucleotide sequence (5’ to 3’)**		**Target**	**Size**	**Reference**
**PCR**				
AB1553	CTCCAATCGGAGGGGTTGTG	*omp6*	492bp	[43]
AB1554	GCATAAATCCAGTTTAGCCTCC			
AB1555	GTGGTTAGATCTTTTCTGTTGGG	*omp7*	399bp	[43]
AB1556	CGCTCTACCACTGACCTTCATG			
AB1591	GCTGGAGTTCGAAGCGATGC	*omp8*	259bp	This study
AB1592	CAGAGCGCCCTGTTTCAGTG			
AB1559	AGCTGGGGCTCTTGCGTTTG	*omp9*	1096bp	[43]
AB1560	AACATATTCACTATAATCTGACGCTGC			
AB1561	TCCTTCGGGTTGCTGCGTTG	*omp10*	969bp	[43]
AB1562	GCTTACCCCCATTCCAGCAC			
AB1572	AGGATGATCAGCCACACTGGAA	*16S*	131bp	This study
AB1573	TACAACCCTAAGGCCTTCCTCA			
**qPCR				
AB1591	GCTGGAGTTCGAAGCGATGC			
AB1592	CAGAGCGCCCTGTTTCAGTG	*omp8*	259bp	This study
AB1593	*GCGTGAGCACTGCGGTACAGACGG*			
AB1581	GAAGTCACTACACGACCTGACTGT			
AB1582	TAAAGCATCTTCGCGGGTCGT	*omp9*	145bp	[43]
AB1583	*TATTCAGTGCGCTGAACACTGCGATCCA*			
AB1594	GTGGGTGCTGTACGCACATT			
AB1595	AAAGACAGCAGGCAGCAACA	*omp10-5'*	170bp	This study
AB1596	*CGCGTGTCCTTCGGGTTGCT*			
AB1569	GGTGCTGAGTTGAAGCTTGC			
AB1570	GCCACAGACCCACTATCAGC	*omp10-3'*	140bp	[43]
AB1571	*TATCTCGCGCTGCATCGGTG*			
AB1572	AGGATGATCAGCCACACTGGAA			
AB1573	TACAACCCTAAGGCCTTCCTCA	*16S*	131bp	[42]
AB1574	*TATTGGACAATGGGCGCAAGCCTGAT*			
AB1606	CTCACAGGCGAAGAAGCAGAC			
AB1607	GCCCGACATACCTGCCTTT	*msp5*	145bp	[55]
AB1610	*TGGGCGACAAGAAGCCAAGTGA*			
AB1608	ATCAAAGCTATTGCGGAGGA			
AB1607	ACAGAACTCTCCCCATGCAC	*rpoH*	116bp	This study
AB1611	*TGCCAATCGGGACGTTTCGC*			
AB1242	AAAACAGGCTTACCGCTCCAA			
AB1243	GGCGTGTAGCTAGGCTCAAAGT	*opag2*	151bp	[41]
AB1250	*CTCTCCTCTGCTCAGGGCTCTGCG*			

*Primers and TaqMan probes used were manufactured at Eurofins MGM Operon.

*Oligonucleotides* are labeled with 6-Carboxyfluorescein 6-FAM at the 5’ end and Tetramethylrhodamine TAMRA at the 3’ end.

### RT-qPCR experiments

Transcript differences between *omp8*, *omp9*, *omp10-5’* end, and *omp10-3*’ genes in *A. marginale* wild-type and *omp10::himar1* mutant were determined using the comparative 2^-∆∆Ct^ method [53,54] and the results were based on the mean of three biological samples (individual RNA extracts). For Taqman quantitative PCR, cDNA obtained from ISE6 cells infected with *A. marginale* wild-type and the *omp10::himar1* was used with primers and probes (Table 1) designed to amplify *omp8*, *omp9*, *omp10-5’* end, *omp10-3*’ end, *msp5*, *rpoH* and the *16S* gene sequences. Reaction conditions are described in Additional file 1: Table S3, specificity of primers and probes is shown in Additional file 1: Figure S1 and the amplification efficiencies for each target are reported in Additional file 1: Table S4. For a valid 2^-∆∆Ct^ calculation, relative efficiencies of target vs. reference genes were calculated and are reported in Additional file 1: Table S5.

Significant differences between the *A. marginale* wild-type and *omp10::himar1* mutant were calculated by Student’s t test (P < 0.05), comparing ∆Ct values (target gene- reference gene) of the *omp10::himar1* mutant and the wild-type. The fold difference was based on ∆∆Ct (*omp10::himar1* mean ∆Ct – wild-type mean ∆Ct) and calculated as 2^-∆∆Ct^ which yields the expression ratio. The expression ratio was then expressed as percentage of expression by multiplying the 2^-∆∆Ct^ values by 100. For normalization of relative gene expression data *msp5*[55], *rpoH*, and *16S* were used as reference genes.

### Western immunoblots

Expression of the Omp9 protein in *A. marginale* wild-type and *omp10*::*himar1* mutant was assessed by sodium dodecyl sulfate-polyacrylamide gel electrophoresis and immunoblotting using equal amounts (10^8^) of host-free bacteria. Membranes were incubated with three different antibodies; the anti-Omp9 monoclonal antibody (121/1055) [43], the monoclonal antibody F16C1 (reacts with the Msp5 protein and served as a loading control) [56] and the monoclonal antibody Tryp1E1 (exhibits specificity for a variable surface glycoprotein of *Trypanosoma brucei*) [56]. This last antibody served as a negative control. Final concentrations of each antibody used were 4 μg/ml, 2 μg/ml and 4 μg/ml. Antibody binding was detected with the secondary antibody goat anti-mouse IgG, horseradish peroxidase labeled and diluted to 1:10,000 using the Pierce ECL Western blotting substrate (Thermo scientific) as described in manufacturer’s instructions.

Quantification of the number of *A. marginale* wild-type and *omp10::himar1* organisms was performed as described elsewhere [41].

### GenBank accession numbers

for assembled contigs containing the *Himar1* transposon sequences integrated within *omp10* and upstream genes (KJ567138) and *omp10* (partial 3’ end) and *omp9* genes (KJ567139).

## Competing interests

The authors declare that they have no competing interests.

## Authors’ contributions

FLC designed and carried out experiments, data analysis and authored this manuscript. HLW and MGP carried out tick cell media preparation, maintenance of uninfected tick cell cultures and co-authored the manuscript. AML performed Western blot experiments and co-authored the manuscript. AFB advised on experiments, genome sequencing analysis, critically evaluated and co-authored the manuscript. SMN provided monoclonal antibodies for Western blot experiments, co-authored and critically evaluated the manuscript. UM kindly provided transformed *A. marginale* organisms co-authored and critically evaluated the manuscript. All authors read and approved the final manuscript.

## Supplementary Material

Additional file 1 RT-PCR and RT-qPCR experiments Figure and Tables. Click here for file

## References

[B1] Kocan KM de la Fuente J Blouin EF Coetzee JF Ewing SA The natural history of *Anaplasma marginale* Vet Parasitol 2010 167 95 107 10.1016/j.vetpar.2009.09.012 19811876

[B2] Kocan KM de la Fuente J Blouin EF Garcia-Garcia JC *Anaplasma marginale* (Rickettsiales: Anaplasmataceae): recent advances in defining host-pathogen adaptations of a tick-borne rickettsia Parasitology 2004 129 Suppl S285 300 15938516 10.1017/s0031182003004700

[B3] Morley RS Hugh-Jones ME The cost of anaplasmosis in the Red River Plains and south-east areas of Louisiana Vet Res Commun 1989 13 349 358 10.1007/BF00346067 2588475

[B4] Goodger WJ Carpenter T Riemann H Estimation of economic loss associated with anaplasmosis in California beef cattle J Am Vet Med Assoc 1979 174 1333 1336 511736

[B5] Palmer GH Brayton KA Antigenic variation and transmission fitness as drivers of bacterial strain structure Cell Microbiol 2013 12 1969 1975 doi:10.1128/CVI.00600-12 23941262 10.1111/cmi.12182 PMC3836861

[B6] Palmer GH Futse JE Knowles DP Jr Brayton KA Insights into mechanisms of bacterial antigenic variation derived from the complete genome sequence of *Anaplasma marginale* Ann N Y Acad Sci 2006 1078 15 25 10.1196/annals.1374.002 17114676

[B7] Meeus PF Brayton KA Palmer GH Barbet AF Conservation of a gene conversion mechanism in two distantly related paralogues of *Anaplasma marginale* Mol Microbiol 2003 47 633 643 10.1046/j.1365-2958.2003.03331.x 12535066

[B8] Meeus PF Barbet AF Ingenious gene generation Trends Microbiol 2001 9 353 355 discussion 355–356 10.1016/S0966-842X(01)02112-6 11514195

[B9] Kocan KM de la Fuente J Guglielmone AA Melendez RD Antigens and alternatives for control of *Anaplasma marginale* infection in cattle Clin Microbiol Rev 2003 16 698 712 10.1128/CMR.16.4.698-712.2003 14557295 PMC207124

[B10] Herndon DR Palmer GH Shkap V Knowles DP Jr Brayton KA Complete genome sequence of *Anaplasma marginale* subsp. centrale J Bacteriol 2010 192 379 380 10.1128/JB.01330-09 19854912 PMC2798241

[B11] Agnes JT Brayton KA LaFollett M Norimine J Brown WC Palmer GH Identification of *Anaplasma marginale* outer membrane protein antigens conserved between *A. marginale* sensu stricto strains and the live *A. marginale* subsp. centrale vaccine Infect Immun 2011 79 1311 1318 10.1128/IAI.01174-10 21189322 PMC3067503

[B12] Dark MJ Al-Khedery B Barbet AF Multistrain genome analysis identifies candidate vaccine antigens of *Anaplasma marginale* Vaccine 2011 29 4923 4932 10.1016/j.vaccine.2011.04.131 21596083 PMC3133685

[B13] de la Fuente J Kocan KM Garcia-Garcia JC Blouin EF Claypool PL Saliki JT Vaccination of cattle with *Anaplasma marginale* derived from tick cell culture and bovine erythrocytes followed by challenge-exposure with infected ticks Vet Microbiol 2002 89 239 251 10.1016/S0378-1135(02)00206-7 12243900

[B14] Rodriguez SD Garcia Ortiz MA, Hernandez Salgado G, Santos Cerda NA, Aboytes Torre R, Canto Alarcon GJ: ***Anaplasma marginale *****inactivated vaccine: dose titration against a homologous challenge** Comp Immunol Microbiol Infect Dis 2000 23 239 252 10.1016/S0147-9571(99)00076-4 11038126

[B15] Kano FS Tamekuni K Coelho AL Garcia JL Vidotto O Itano EN Vidotto MC Induced immune response of DNA vaccine encoding an association MSP1a, MSP1b, and MSP5 antigens of *Anaplasma marginale* Vaccine 2008 26 3522 3527 10.1016/j.vaccine.2008.04.047 18502005

[B16] Brown WC Shkap V Zhu D McGuire TC Tuo W McElwain TF Palmer GH CD4^(+)^ T-lymphocyte and immunoglobulin G2 responses in calves immunized with *Anaplasma marginale* outer membranes and protected against homologous challenge Infect Immun 1998 66 5406 5413 9784551 10.1128/iai.66.11.5406-5413.1998 PMC108677

[B17] Brown WC Zhu D Shkap V McGuire TC Blouin EF Kocan KM Palmer GH The repertoire of *Anaplasma marginale* antigens recognized by CD4(+) T-lymphocyte clones from protectively immunized cattle is diverse and includes major surface protein 2 (MSP-2) and MSP-3 Infect Immun 1998 66 5414 5422 9784552 10.1128/iai.66.11.5414-5422.1998 PMC108678

[B18] Noh SM Brayton KA Brown WC Norimine J Munske GR Davitt CM Palmer GH Composition of the surface proteome of *Anaplasma marginale* and its role in protective immunity induced by outer membrane immunization Infect Immun 2008 76 2219 2226 10.1128/IAI.00008-08 18316389 PMC2346715

[B19] Noh SM Turse JE Brown WC Norimine J Palmer GH Linkage between *Anaplasma marginale* outer membrane proteins enhances immunogenicity but is not required for protection from challenge Clin Vaccine Immunol 2013 20 651 656 10.1128/CVI.00600-12 23446216 PMC3647754

[B20] Sutten EL Norimine J Beare PA Heinzen RA Lopez JE Morse K Brayton KA Gillespie JJ Brown WC *Anaplasma marginale* type IV secretion system proteins VirB2, VirB7, VirB11, and VirD4 are immunogenic components of a protective bacterial membrane vaccine Infect Immun 2010 78 1314 1325 10.1128/IAI.01207-09 20065028 PMC2825951

[B21] Brayton KA Kappmeyer LS Herndon DR Dark MJ Tibbals DL Palmer GH McGuire TC Knowles DP Jr Complete genome sequencing of *Anaplasma marginale* reveals that the surface is skewed to two superfamilies of outer membrane proteins Proc Natl Acad Sci U S A 2005 102 844 849 10.1073/pnas.0406656102 15618402 PMC545514

[B22] Palmer GH Brown WC Noh SM Brayton KA Genome-wide screening and identification of antigens for rickettsial vaccine development FEMS Immunol Med Microbiol 2012 64 115 119 10.1111/j.1574-695X.2011.00878.x 22066488 PMC3288579

[B23] Dark MJ Lundgren AM Barbet AF Determining the repertoire of immunodominant proteins via whole-genome amplification of intracellular pathogens PLoS One 2012 7 e36456 doi:10.1371/journal.pone.0036456 10.1371/journal.pone.0036456 22558468 PMC3340345

[B24] Brayton KA Palmer GH Brown WC Genomic and proteomic approaches to vaccine candidate identification for *Anaplasma marginale* Expert Rev Vaccines 2006 5 95 101 10.1586/14760584.5.1.95 16451111

[B25] Pierle SA Dark MJ Dahmen D Palmer GH Brayton KA Comparative genomics and transcriptomics of trait-gene association BMC Genomics 2012 13 669 10.1186/1471-2164-13-669 23181781 PMC3542260

[B26] Van Opijnen T Camilli A Transposon insertion sequencing: a new tool for systems-level analysis of microorganisms Nat Rev Microbiol 2013 11 435 442 10.1038/nrmicro3033 23712350 PMC3842022

[B27] Claeys Bouuaert C Chalmers RM Gene therapy vectors: the prospects and potentials of the cut-and-paste transposons Genetica 2010 138 473 484 10.1007/s10709-009-9391-x 19649713

[B28] Picardeau M Transposition of fly mariner elements into bacteria as a genetic tool for mutagenesis Genetica 2010 138 551 558 10.1007/s10709-009-9408-5 19757097

[B29] Lampe DJ Grant TE Robertson HM Factors affecting transposition of the *Himar1* mariner transposon in vitro Genetics 1998 149 179 187 9584095 10.1093/genetics/149.1.179 PMC1460121

[B30] Plasterk RH Izsvak Z Ivics Z Resident aliens: the Tc1/mariner superfamily of transposable elements Trends Genet 1999 15 326 332 10.1016/S0168-9525(99)01777-1 10431195

[B31] Clark TR Ellison DW Kleba B Hackstadt T Complementation of *Rickettsia rickettsii* RelA/SpoT restores a nonlytic plaque phenotype Infect Immun 2011 79 1631 1637 10.1128/IAI.00048-11 21300770 PMC3067566

[B32] Clark TR Lackey AM Kleba B Driskell LO Lutter EI Martens C Wood DO Hackstadt T Transformation frequency of a mariner-based transposon in *Rickettsia rickettsii* J Bacteriol 2011 193 4993 4995 10.1128/JB.05279-11 21764933 PMC3165637

[B33] Kleba B Clark TR Lutter EI Ellison DW Hackstadt T Disruption of the R*ickettsia rickettsii* Sca2 autotransporter inhibits actin-based motility Infect Immun 2010 78 2240 2247 10.1128/IAI.00100-10 20194597 PMC2863521

[B34] Beare PA Sandoz KM Omsland A Rockey DD Heinzen RA Advances in genetic manipulation of obligate intracellular bacterial pathogens Front Microbiol 2011 2 97 21833334 10.3389/fmicb.2011.00097 PMC3153054

[B35] Beare PA Genetic manipulation of *Coxiella burnetii* Adv Exp Med Biol 2012 984 249 271 10.1007/978-94-007-4315-1_13 22711636

[B36] Botkin DJ Abbott AN Stewart PE Rosa PA Kawabata H Watanabe H Norris SJ Identification of potential virulence determinants by *Himar1* transposition of infectious *Borrelia burgdorferi* B31 Infect Immun 2006 74 6690 6699 10.1128/IAI.00993-06 17015459 PMC1698074

[B37] Stewart PE Hoff J Fischer E Krum JG Rosa PA Genome-wide transposon mutagenesis of *Borrelia burgdorferi* for identification of phenotypic mutants Appl Environ Microbiol 2004 70 5973 5979 10.1128/AEM.70.10.5973-5979.2004 15466540 PMC522107

[B38] Maier TM Casey MS Becker RH Dorsey CW Glass EM Maltsev N Zahrt TC Frank DW Identification of *Francisella tularensis Himar1*-based transposon mutants defective for replication in macrophages Infect Immun 2007 75 5376 5389 10.1128/IAI.00238-07 17682043 PMC2168294

[B39] Cheng C Nair AD Indukuri VV Gong S Felsheim RF Jaworski D Munderloh UG Ganta RR Targeted and random mutagenesis of *Ehrlichia chaffeensis* for the identification of genes required for in vivo infection PLoS Pathog 2013 9 e1003171 doi:10.1371/journal.ppat.1003171 10.1371/journal.ppat.1003171 23459099 PMC3573109

[B40] Felsheim RF Herron MJ Nelson CM Burkhardt NY Barbet AF Kurtti TJ Munderloh UG Transformation of *Anaplasma phagocytophilum* BMC Biotechnol 2006 6 42 10.1186/1472-6750-6-42 17076894 PMC1635035

[B41] Felsheim RF Chavez AS Palmer GH Crosby L Barbet AF Kurtti TJ Munderloh UG Transformation of *Anaplasma marginale* Vet Parasitol 2010 167 167 174 10.1016/j.vetpar.2009.09.018 19837516 PMC2817780

[B42] Barbet AF Agnes JT Moreland AL Lundgren AM Alleman AR Noh SM Brayton KA Munderloh UG Palmer GH Identification of functional promoters in the *msp2* expression loci of *Anaplasma marginale* and *Anaplasma phagocytophilum* Gene 2005 353 89 97 10.1016/j.gene.2005.03.036 15935572

[B43] Noh SM Brayton KA Knowles DP Agnes JT Dark MJ Brown WC Baszler TV Palmer GH Differential expression and sequence conservation of the *Anaplasma marginale msp2* gene superfamily outer membrane proteins Infect Immun 2006 74 3471 3479 10.1128/IAI.01843-05 16714578 PMC1479288

[B44] Guell M van Noort V Yus E Chen WH Leigh-Bell J Michalodimitrakis K Yamada T Arumugam M Doerks T Kuhner S Rode M Suyama M Schmidt S Gavin AC Bork P Serrano L Transcriptome complexity in a genome-reduced bacterium Science 2009 326 1268 1271 10.1126/science.1176951 19965477

[B45] Kleckner N Translocatable elements in procaryotes Cell 1977 11 11 23 10.1016/0092-8674(77)90313-0 326413

[B46] Beare PA Gilk SD Larson CL Hill J Stead CM Omsland A Cockrell DC Howe D Voth DE Heinzen RA Dot/Icm type IVB secretion system requirements for *Coxiella burnetii* growth in human macrophages MBio 2011 2 e00175 00111 21862628 10.1128/mBio.00175-11 PMC3163939

[B47] Lopez JE Siems WF Palmer GH Brayton KA McGuire TC Norimine J Brown WC Identification of novel antigenic proteins in a complex *Anaplasma marginale* outer membrane immunogen by mass spectrometry and genomic mapping Infect Immun 2005 73 8109 8118 10.1128/IAI.73.12.8109-8118.2005 16299305 PMC1307060

[B48] Riding G Hope M Waltisbuhl D Willadsen P Identification of novel protective antigens from *Anaplasma marginale* Vaccine 2003 21 1874 1883 10.1016/S0264-410X(03)00004-5 12706672

[B49] Munderloh UG Blouin EF Kocan KM Ge NL Edwards WL Kurtti TJ Establishment of the tick (Acari:Ixodidae)-borne cattle pathogen *Anaplasma marginale* (Rickettsiales:Anaplasmataceae) in tick cell culture J Med Entomol 1996 33 656 664 8699463 10.1093/jmedent/33.4.656

[B50] Munderloh UG FR Burkhardt NY Herron MJ Olivia Chavez AS Nelson CM Kurtti TJ Azad A, Palmer G The way forward: improving genetic systems Intracellular Pathogens II 2012 Washington, DC: ASM Press 416 432

[B51] Shanner N Campbell RE Steinbach PA Giepmans BN Palmer AE Tsien RY Improved monomeric red, orange and yellow fluorescent proteins derived from Discosoma sp. red fluorescent protein Nat Biotechnol 2004 22 5 10.1038/nbt0104-5 15558047

[B52] Qin A Tucker AM Hines A Wood DO Transposon mutagenesis of the obligate intracellular pathogen *Rickettsia prowazekii* Appl Environ Microbiol 2004 70 2816 2822 10.1128/AEM.70.5.2816-2822.2004 15128537 PMC404435

[B53] Schmittgen TD Livak KJ Analyzing real-time PCR data by the comparative C(T) method Nat Protoc 2008 3 1101 1108 10.1038/nprot.2008.73 18546601

[B54] Livak KJ Schmittgen TD Analysis of relative gene expression data using real-time quantitative PCR and the 2^-ΔΔCt^ Method Methods 2001 25 402 408 10.1006/meth.2001.1262 11846609

[B55] Lohr CV Rurangirwa FR McElwain TF Stiller D Palmer GH Specific expression of *Anaplasma marginale* major surface protein 2 salivary gland variants occurs in the midgut and is an early event during tick transmission Infect Immun 2002 70 114 120 10.1128/IAI.70.1.114-120.2002 11748171 PMC127638

[B56] Barbet AF Blentlinger R Yi J Lundgren AM Blouin EF Kocan KM Comparison of surface proteins of *Anaplasma marginale* grown in tick cell culture, tick salivary glands, and cattle Infect Immun 1999 67 102 107 9864202 10.1128/iai.67.1.102-107.1999 PMC96283

