# Supplementary material for: Knockout of an outer membrane protein operon of Anaplasma marginale by transposon mutagenesis
Source: BMC Genomics. 2014 Apr 11;15:278. doi: 10.1186/1471-2164-15-278 (PMC4198910; doi:10.1186/1471-2164-15-278)
Supplement: Additional file 1 — RT-PCR and RT-qPCR experiments Figure and Tables. [file 1471-2164-15-278-S1.pdf]

Figure 1. **Specificity of primers and probes in RT-qPCR reactions.**

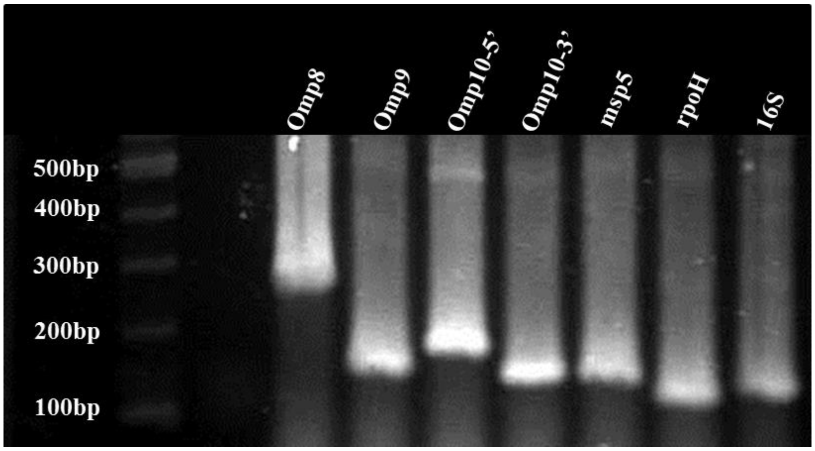

To evaluate the target specificity of primers and probes 5μl of qPCR product from each target was analyzed in a 2% Seakem LE (Lonza) agarose gel stained with SYBR gold nucleic acid gel stain (Life-Technologies), (*omp8*, 259bp), (*omp9*, 145bp), (*omp10-5*' end, 170bp), (*omp10-3*' end, 140bp), (*msp5*, 139bp), (*rpoH*, 116bp) and (*16S*, 131bp).

Table 1. PCR conditions for the amplification of transcripts from *omp7* through *AM1225* intergenic regions.

	Step	Temperature	Time
	Denaturation	94°C	2 minutes
35 cycles	Denaturation	94°C	10 seconds
	Annealing	65°C	30 seconds
	Extension	68°C	15 seconds
	Extension	68°C	5 minutes

PCR reactions were performed in Bio-Rad PTC-200 thermocycler. Each reaction contained 200ng of cDNA combined with 200µM dNTP's, 0.3µM of forward and reverse primers and 1.25 units of primeSTAR GXL DNA polymerase (Takara). PCR products were electrophoretically separated using a 1 and 2% Seakem LE (Lonza) Agarose gel, and stained with SYBR gold nucleic acid gel stain (Life technologies) for UV visualization.

Table 2. PCR conditions for the amplification of transcripts from *omp6* through *omp10* from cDNA obtained from ISE6 cells infected with *A. marginale* wild-type and *omp10::himar1* mutant organisms.

	Step	Temperature	Time
	Denaturation	94°C	2 minutes
35 cycles	Denaturation	94°C	10 seconds
35 cycles	Annealing	*60°C/**65°C	58 seconds
35 cycles	Extension	68°C	2 minutes
	Extension	68°C	10 minutes

PCR reactions were performed in Bio-Rad PTC-200 thermocycler.

*Annealing temperatures used for the amplification of *omp6*, *omp7*, *omp8* and *16S* ribosomal subunit.

**Annealing temperature used for the amplification of *omp9* and *omp10*.

Each reaction contained 200ng of cDNA combined with 200µM dNTP's, 0.3µM of forward and reverse primers and 1.25 units of primeSTAR GXL DNA polymerase (Takara). PCR products were electrophoretically separated using a 1 and 2% Seakem LE (Lonza) Agarose gel, and stained with SYBR gold nucleic acid gel stain (Life technologies) for UV visualization.

Table 3. **qPCR reactions and amplification conditions.**

	Step	Temperature	Time
	Denaturation	95°C	15 minutes
40 cycles	Denaturation	94°C	15 seconds
40 cycles	Annealing/Extension	60°C	1 minute
40 cycles	Data collection		

Each reaction had a net volume of 25μl containing 5μl of cDNA, 1X Quantitect Probe PCR master mix (QIAGEN), 0.4μM of forward and reverse primers, and 0.2μM of probe.

Table 4. qPCR amplification efficiencies.

*GOI	qPCR efficiency $E=(5^{-1/slope})-1$
omp8	0.98
omp9	0.96
omp10-5' end	0.93
omp10-3' end	0.97
msp5	0.95
rpoH	0.90
16S	0.90

Primer-probe reaction efficiencies were determined for each primer-probe set targeting *omp8*, *omp9*, *omp10*-3' end, *omp10-5*' end, *msp5*, *rpoH*, and *16S* rRNA by performing triplicate reactions of a ten log range of 5-fold serial dilutions of cDNA for the purpose of generating standard curves from which reaction efficiencies could be calculated [1, 2].

*GOI, gene of interest.

Table 5. Relative amplification efficiencies.

Reference genes	Relative efficiencies slopes	
	omp8	0 .0327
msp5	*omp9*	0 .0182
	omp10-5' end	0 .0267
	omp10-3' end	0 .0826
	omp8	0 .0273
rpoH	*omp9*	0 .0127
	omp10-5' end	0 .0676
	omp10-3' end	0 .1211
	omp8	0 .0226
16S	*omp9*	0 .0848
	omp10-5' end	0 .0061
	omp10-3' end	0 .0223

Relative amplification efficiencies of the target genes (*omp8, 9, 10-5'* end and 10-3' end) versus reference genes (*msp5, rpoH,* and *16S*) were calculated by performing standard curves for each amplicon in the same manner and conditions to those used to determine the amplification efficiencies. The Ct values generated were used to calculate a ΔCt value (Ct target gene-Ct reference gene) for each sample which corrected for any template loading discrepancy between reactions. Plots of ΔCt values versus the log of cDNA input were used to determine the slope of standard curves [1, 2].

REFERENCES.

1. Bustin SA, Benes V, Garson JA, Hellemans J, Huggett J, Kubista M, Mueller R, Nolan T, Pfaffl MW, Shipley GL *et al*: **The MIQE guidelines: minimum information for publication of quantitative real-time PCR experiments**. *Clin Chemy* 2009, **55**: 611-622.
2. Tuzmen S, Kiefer J, Mousses S: **Validation of short interfering RNA knockdowns by quantitative real-time PCR**. *Methods Mol Biol* 2007, **353**:177-203.
